# Conversion of Normal Ly-1-Positive B-Lineage Cells into Ly-1-Positive Macrophages in Long-Term Bone Marrow Cultures

**DOI:** 10.1155/1990/28760

**Published:** 1990

**Authors:** Shigeki Katoh, Akira Tominaga, Masahiro Migita, Akira Kudo, Kiyoshi Takatsu

**Affiliations:** 1 Department of Biology Institute for Medical Immunology Kumamoto University Medical School 2-2-1 Honjo Kumamoto 860 Japan; 2 Basel Institute for Immunology Grenzacherstrasse Basel CH-4000 Switzerland

## Abstract

We obtained eight different cell lines in the long-term bone marrow culture system that showed a germ-line configuration of the joining (J) region segments of the Ig heavy-chain (IgH) genes. Their surface markers were CD45R^+^, Ly-1^+^, Lyb-2^+^, cIgM^-^, sIgM^-^, Ia^-^, Thy-1^-^, Mac-1^-^, and IL-2R (Tac)^+^. Use of very young mice and the presence of IL-5 were important for preferential promotion of the survival of B-lineage lymphocytes bearing the Ly-1 markers. When we treated two of them (J8 and J10) with 5-azacytidine for 24 h followed by co-culture with stromal cells and IL-.5, they became Ly-1^+^, sIgM^+^ B cells, and Ly-1^+^, Mac-1^+^ macrophagelike cells, respectively. After other early lymphoid lines (J1, J8, and J13) were maintained by co-culture with ST2 and IL-5 for more than a year, they showed a heterogeneous DNA rearrangement profile of the J region segment of the IgH gene, although only J13 rearranged the κ-light chain gene. Northern blot analysis revealed that these cell lines expressed Cμ-mRNA, and λ5-mRNA, consistent with normal pre-B cells. Intriguingly, J1, J8, and J13 expressed c-*fms* mRNA constitutively. When J13 cells were co-cultured with ST2 and GM-CSF in place of ST2 and IL-5, they acquired Mac-1 expression and retained Ly-1 expression. They were morphologically macrophages, nonspecific-esterase-positive, and showed phagocytosis of latex beads. These results support evidence for a close relationship between the myeloid and Ly-1^+^ B-cell pathways of differentiation, and indicate that our IL- 5-dependent clones are multipotential intermediates in differentiation from pro-B cells to B cells and macrophages.


*Developmental Immunology*, 1990, Vol. 1, pp. 113–125
Reprints available directly from the publisher
Photocopying permitted by license only

© 1990 Harwood Academic Publishers GmbH
Printed in the United Kingdom

### Conversion of Normal Ly-1-Positive B-Lineage Cells into Ly-1-Positive Macrophages in Long-Term Bone Marrow Cultures

SHIGEKI KATOH†, AKIRA TOMINAGA,† MASAHIRO MIGITA,† AKIRA KUDO‡ and KIYOSHI TAKATSU*†

†*Department of Biology, Institute for Medical Immunology, Kumamoto University Medical School, 2-2-1 Honjo, Kumamoto 860, Japan*
‡*Basel Institute for Immunology, Grenzacherstrasse, CH-4000, Basel, Switzerland*

We obtained eight different cell lines in the long-term bone marrow culture system that showed a germ-line configuration of the joining (J) region segments of the Ig heavy-chain (IgH) genes. Their surface markers were CD45R$^+$, Ly-1$^+$, Lyb-2$^+$, cIgM$^-$, sIgM$^-$, Ia$^-$, Thy-1$^-$, Mac-1$^-$, and IL-2R (Tac)$^+$. Use of very young mice and the presence of IL-5 were important for preferential promotion of the survival of B-lineage lymphocytes bearing the Ly-1 markers. When we treated two of them (J8 and J10) with 5-azacytidine for 24 h followed by co-culture with stromal cells and IL-5, they became Ly-1$^+$, sIgM$^+$ B cells, and Ly-1$^+$, Mac-1$^+$ macrophagelike cells, respectively. After other early lymphoid lines (J1, J8, and J13) were maintained by co-culture with ST2 and IL-5 for more than a year, they showed a heterogeneous DNA rearrangement profile of the J region segment of the IgH gene, although only J13 rearranged the $\kappa$-light chain gene. Northern blot analysis revealed that these cell lines expressed C$\mu$-mRNA, and $\lambda$5-mRNA, consistent with normal pre-B cells. Intriguingly, J1, J8, and J13 expressed c-*fms* mRNA constitutively. When J13 cells were co-cultured with ST2 and GM-CSF in place of ST2 and IL-5, they acquired Mac-1 expression and retained Ly-1 expression. They were morphologically macrophages, nonspecific-esterase-positive, and showed phagocytosis of latex beads. These results support evidence for a close relationship between the myeloid and Ly-1$^+$ B-cell pathways of differentiation, and indicate that our IL-5-dependent clones are multipotential intermediates in differentiation from pro-B cells to B cells and macrophages.

KEYWORDS: Long-term bone marrow culture, IL-5, Ly-1$^+$ cells, pre-B cells, macrophages, c-*fms*.

### INTRODUCTION

B lymphocytes derive from hematopoietic stem cells in bone marrow that also generates myeloid and erythroid elements, but decisive commitment to the lymphoid lineage is championed by deletions that create a functional immunuglobulin (Ig) heavy-chain variable gene (Kincade, 1987). These deletions occur at a defined stage of lymphoid ontogeny and are characteristic of each lymphocyte clone. Intriguingly, from several tumors bearing a B-lineage marker, it has been possible to obtain cells with myeloid characteristics, such as macrophages (Boyd and Schrader, 1982; Bauer et al., 1986; Holmes et al., 1986). Conversely, Ig gene rearrangements have recently been detected in a subset of acute myelogenous leukemias (Seremitis et al., 1987). Furthermore, cloned pre-B- and B-cell lines from bone marrow cells in E$\mu$-*myc* transgenic mice infected with a retrovirus bearing v-*raf* changed into either mature or immature macrophages, but retained clonotypic Ig gene rearrangements (Klinken et al., 1988). One interpretation of such observations is that the B and myeloid lineages are unexpectedly close (Holmes et al., 1986; Davidson et al., 1988).

IL-5 has been defined as a growth and differentiation factor acting on mature murine B cells (Kinashi et al., 1986; Coffman et al., 1988; Takatsu et al., 1988). Studies on functional properties of rIL-5 revealed that IL-5 acts at least on B cells, T cells, and eosinophils, inducing their growth and differentiation (Takatsu et al., 1987; Yokota et al., 1987;

*Corresponding author.

Sanderson et al., 1988; Coffman et al., 1989). We also reported that IL-5 is at least one growth factor that can sustain growth of Ly-1 positive B-lineage cell lines (Tominaga et al., 1989). However, the role of IL-5 in proliferation and differentiation of progenitor cells for macrophages is still not clear.

We have now extended our previous studies and established eight different IL-5-dependent Ly-1$^+$ and CD45R$^+$ cell lines whose IgH gene configurations are germ-line, and determined their potentials for differentiation into B cells or macrophages. Both Ly-1$^+$ early B-lineage cells and Ly-1$^+$ pre-B cells differentiated into not only Ly-1$^+$ B cells, but also Ly-1$^+$ macrophages when co-cultured with bone marrow stromal cells and IL-5 and/or GM-CSF. The results support earlier evidence for a close relationship between the myeloid and certain B-cell pathways of differentiation. They also indicate that such IL-5-dependent clones are useful tools for studying mechanisms of differentiation of multipotential progenitor cells into B cells and macrophages.

### RESULTS

### Derivation and Characterization of IL-5- and Stromal-Cell-Dependent Cell Lines

We first compared the appearance of Ly-1$^+$ B-lineage cells in the original Whitlock–Witte type long-term bone marrow culture (LTBC-W) (Whitlock and Witte, 1982) of 3-week-old bone marrow cells or 8-week-old bone marrow cells of DBA/2J mice. After 4 weeks, we characterized the appearance of Ly-1 and CD45R antigens. As shown in Fig. 2A, cell growth of a distinct Ly-1$^+$ and CD45R$^+$ B-lineage

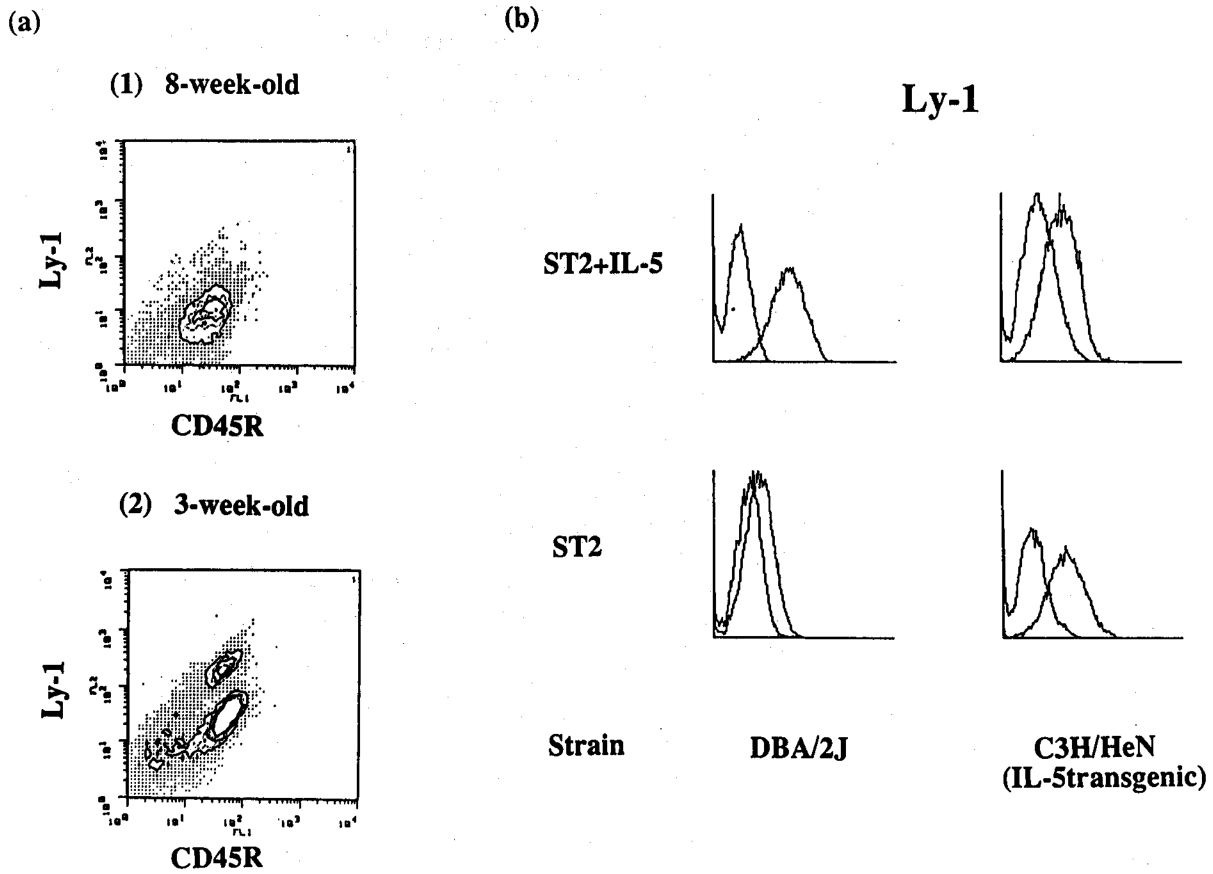

FIGURE 1. Phenotypic characteristics of the Ly-1 clones. (A) Bone marrow cells of 3-week-old or 8-week-old DBA/2J mice were cultured in LTBC-W for 4 weeks. (B) Bone marrow cells from DBA/2J (3-week-old) or IL-5 transgenic C3H/HeN (3-week-old) mice were cultured in LTBC-W for 5 weeks. Nonadherent cells were then transferred onto the ST2 layer and cultured with or without IL-5 (50 U/ml) for 5 weeks. Cells thus obtained were stained with FITC-conjugated anti-Ly-1 and anti-CD45R plus FITC-coupled anti-rat $\kappa$ and were analyzed by a flow cytometry. In the case of (A), biotinylated anti-Ly-1 plus PE-avidin were used instead of FITC-conjugated antibody. Negative controls were cells unstained or stained with the second-step reagent only.

cell population became evident in LTBC-W of 3-week-old bone marrow cells. In contrast, there was very little Ly-1$^+$ population, if any, in LTBC-W of 8-week-old bone marrow cells.

To evaluate the role of ST2 and IL-5 in the establishment of a Ly-1$^+$ B-lineage cell line, we cultured bone marrow cells from 3-week-old DBA/2J mice or from 3-week-old IL-5 transgenic (C3H/HeN) mice whose bone marrow cells constitutively produce IL-5 (Tominaga et al., 1989). After 5 weeks, nonadherent cells of each culture were transferred onto the stromal cell (ST2) layer (Ogawa et al., 1988) and cultured in the presence or absence of IL-5 for another 5 weeks. In DBA/2J bone marrow cells, preferential induction of Ly-1$^+$ B-lineage cells was observed only in the presence of IL-5 (Fig. 1B). In contrast, Ly-1$^+$ B-lineage cells were induced in LTBC-W of bone marrow cells from the IL-5 transgenic mice without adding exogenous IL-5.

Among 18 Ly-1$^+$ clones analyzed in this study, eight clones showed the germ-line type IgH genes, two clones had rearranged IgH genes in both chromosomes, and eight clones displayed one rearranged IgH gene. All cell lines expressed CD45R and Ly-1 antigens. In addition, they were cIgM$^-$, sIgM$^-$, Mac-1$^-$, Ia$^-$, Thy-1$^-$, and IL-2R(Tac)$^+$. On the basis of the foregoing phenotypic and molecular criteria, these cell lines were equivalent to early B-lineage progenitors. Five clones (J1, J2, J8, J10, and J13) having a germ-line configuration of IgH genes were selected and used for further analysis to explore their differentiation potential.

### Induction of Macrophages from B-lineage Cell Lines

We maintained five clones described in the previous section on the ST2 layer with IL-5. Three months later, all cell lines still showed a germ-line configuration of IgH gene, except J8, which showed a rearranged pattern of IgH genes. At this stage, we did not note any change in cell-surface markers from the parental clones (data not shown).

It has been reported that exposure of pre-B lymphoma line to 5-azacytidine induced derivation of macrophagelike lines (Boyd and Schrader, 1982). We therefore cultured J8 and J10 cells in the presence of various concentrations of 5-azacytidine. Of cells cultured with 5 $\mu$g/ml 5-azacytidine, fed after 24 h with fresh medium containing IL-5, and co-cultured on the ST2 layer, only 10% of the initial number were present after 7 days, and most of the cells died within 2 weeks after the treatment. Some of them started to grow after 3 weeks of treatment. After 5 weeks, nonadherent cells (J8A and J10A) were transferred into a flask with fresh ST2 layers and maintained for 3 months in the presence of IL-5. Southern blot analysis of the J8A DNA revealed that the configuration of $J_H$ gene sequences was rearranged, whereas J10A cells showed a germ-line-type configuration like the parental cell line (J10) (data not shown). A flow cytometry analysis revealed that J8A cells were sIgM$^+$, Ly-1$^+$, and Mac-1$^-$, and J10A cells were sIgM$^-$, Ly-1$^+$, and Mac-1$^+$ (Fig. 2). The J8A cells exhibited a characteristic lymphocyte morphology. They were round, medium to small in size, and had large nuclei with scanty cytoplasm devoid of cytoplasmic granules. In contrast, J10A cells were significantly larger, irregularly shaped, and had a large pale cytoplasm riddled with vacuoles-cytological features typical of macrophages (Fig. 3B) and distinctly different from parental J10 cells (Fig. 3A). They were also nonspecific esterase-positive (Fig. 3D) and acquired the ability to engulf latex particles (data not shown), indicating that J10A cells have distinctive properties of mature macrophages. J8A cells were nonspecific esterase-negative (Fig. 3C). IL-5 was essential for the conversion of pro-B cells (J8) to pre-B cells (J8A), but it was not essential for the conversion of pro-B cells (J10) to macrophagelike cells (J10A) (data not shown).

### Establishment of Stromal Cell and IL-5-Dependent Pre-B Cells

Pre-B cell lines were also developed when the original clones were maintained for more than a year on the ST2 layer in the presence of IL-5. After isolating J1, J8, and J13 cells on the ST2 monolayer in the presence of IL-5, we continued the culture by transferring nonadherent cells from flask to flask with newly prepared ST2 monolayers every 3 weeks. During culture in the same flask, cells were fed with fresh medium supplemented with IL-5 every 3 to 4 days. J1, J8, and J13 cells were CD45R$^+$, Ly-1$^+$, and Mac-1$^-$. Southern blot analysis revealed that all cell lines thus obtained displayed rearranged IgH gene configuration (Fig. 4A and 4B), and only the J13 cell line showed $J_\kappa$ rearrangement (Fig. 4C).

To determine whether C$\mu$ transcripts were produced in early B-cell progenitors, Northern blots of total RNA extracted from the panel of clones were hybridized with a C$\mu$ probe. Two other pre-B cell lines (70Z/3 and MJ88-1) and one mature B-cell line

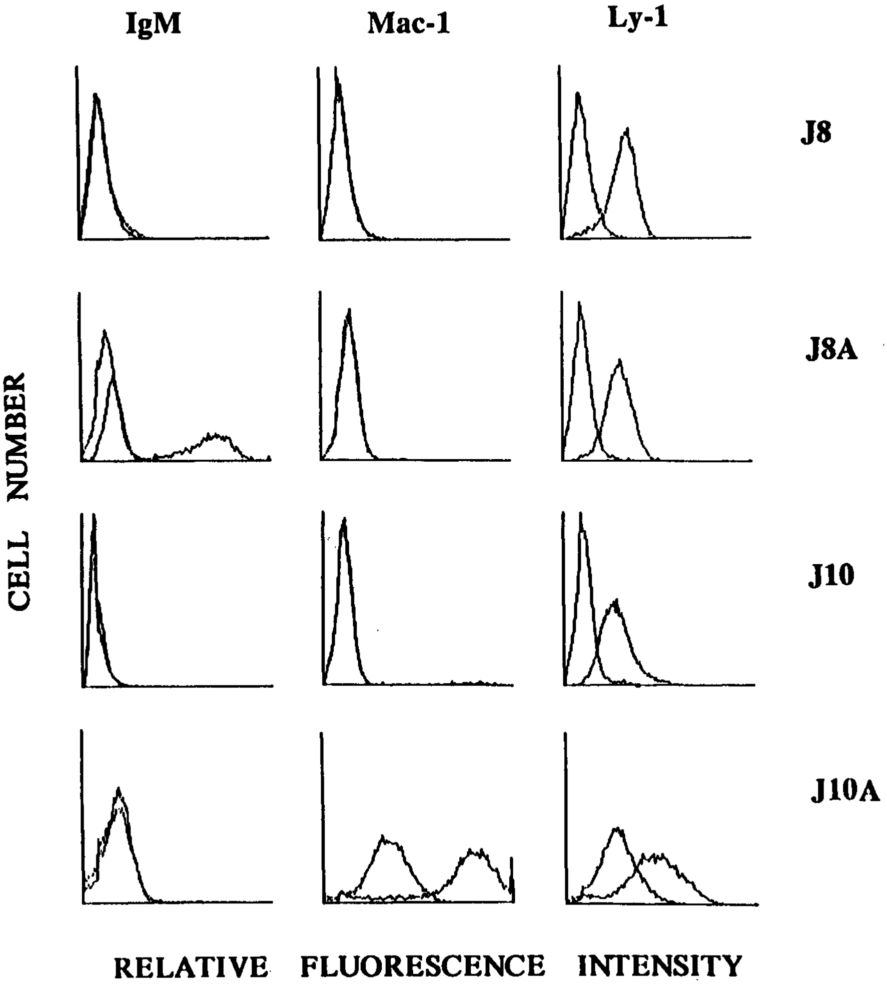

FIGURE 2. Phenotype characteristics of early B-lineage cell lines treated with 5-azacytidine. The clones were treated with 5-azacytidine as detailed in Materials and Methods. Cells obtained 5 weeks after completion of the 5-azacytidine treatment were stained with FITC-coupled anti-$\mu$, FITC-coupled anti-Ly-1, or anti-Mac-1 plus FITC-coupled anti-rat $\kappa$ and analyzed by a flow cytometer.

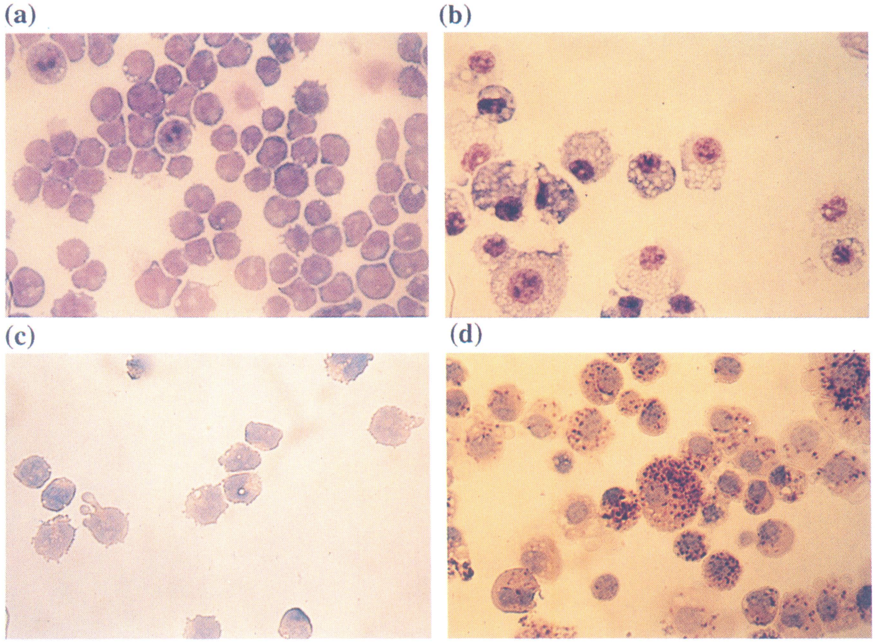

FIGURE 3. Morphological and cytochemical analysis of the J10 clone and its derivative J10A. Part (A) shows cytocentrifuged J10 cells stained with May-Gruenwald-Giemsa solution, and (B) shows cytocentrifuged macrophage-converted J10A cells stained with May-Gruenwald-Giemsa solution. Part (C) shows J8A cells stained with nonspecific esterase, and (D) shows J10A cells stained with nonspecific esterase. The magnification in each case was 400×. (See Colour Plate IV at the back of this publication.)

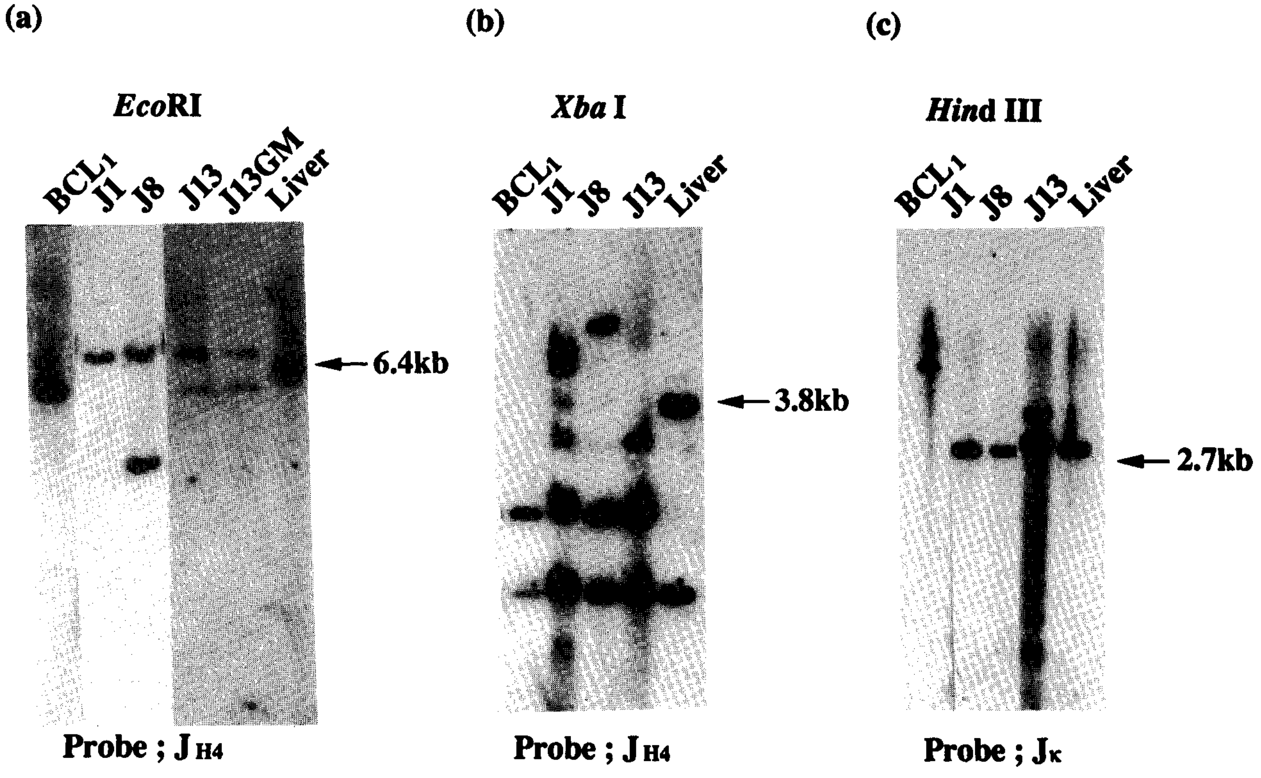

FIGURE 4. Southern blot analysis of pre-B cell lines. DNA of various cell lines were digested with (A) *Eco*RI, (B) *Xba*I, or (C) *Hind*III. Digested DNA were analyzed by Southern blot using (A and B) $J_{H4}$ probe or (C) $J_k$ probe.

($BCL_1$-B20) were used as controls. MJ88-1 is an IL-5-independent and ST2-dependent pre-B cell line (Migita et al., in press). Expression of $\beta$-actin was also examined to normalize amounts of mRNA for the analysis (Fig. 5D). As can be seen in Fig. 5A, J1, J8, and J13 showed 2.7 kb mRNA corresponding to membrane-type $\mu$-chain, although the expression was weaker than in 70Z/3 and MJ88-1 cells. Intriguingly, J1, J8, and J13 as well as 70Z/3 expressed abundant mRNA for $\lambda$5 (Fig. 5B), whereas $BCL_1$ did not express $\lambda$5 mRNA. The MJ88-1 cell line also expressed abundant $\lambda$5 mRNA. To our surprise, abundant c-*fms* mRNA was observed in all IL-5- and ST2-dependent pre-B cell lines, whereas IL-5-independent cell lines such as MJ88-1 and 70Z/3 did not express c-*fms* mRNA (Fig. 5C). Small percentages (5–10%) of these cell lines expressed sIgM (Fig. 6), but neither surface $\kappa$- nor $\lambda$-light chain expression was detectable. The lines were able to grow only in the presence of 2-ME, and required ST2 in addition to IL-5 for their growth.

**Induction of Macrophages from Pre-B Cell Lines**

We co-cultured the pre-B cell line J13 with ST2 plus GM-CSF in place of ST2 plus IL-5, because c-*fms* has been shown to encode receptors for M-CSF (Sherr et al., 1985) and plays a role in monocyte differentiation (Sariban et al., 1985). After 3 weeks of culture, we found growth of Ly-1$^+$ cells (J13GM) with macrophage morphology that had lost surface $\mu$-chain and acquired Mac-1 expression (Fig. 7). Furthermore, they were nonspecific esterase-positive (Fig. 8A) and could ingest latex beads (Fig. 8B). A converted myeloid line (J13GM) had the same $J_H$ rearrangement pattern as its lymphoid parent (J13) (Fig. 4A). In the absence of ST2, it was difficult to obtain macrophagelike cells and a conditioned medium of ST2 could not replace the ST2 adherent layer in this culture system. Multiple tests confirmed that the lines were indeed myeloid and delineated their stage of maturation. Converted cells had twice the doubling time of the parental lines, an obser-

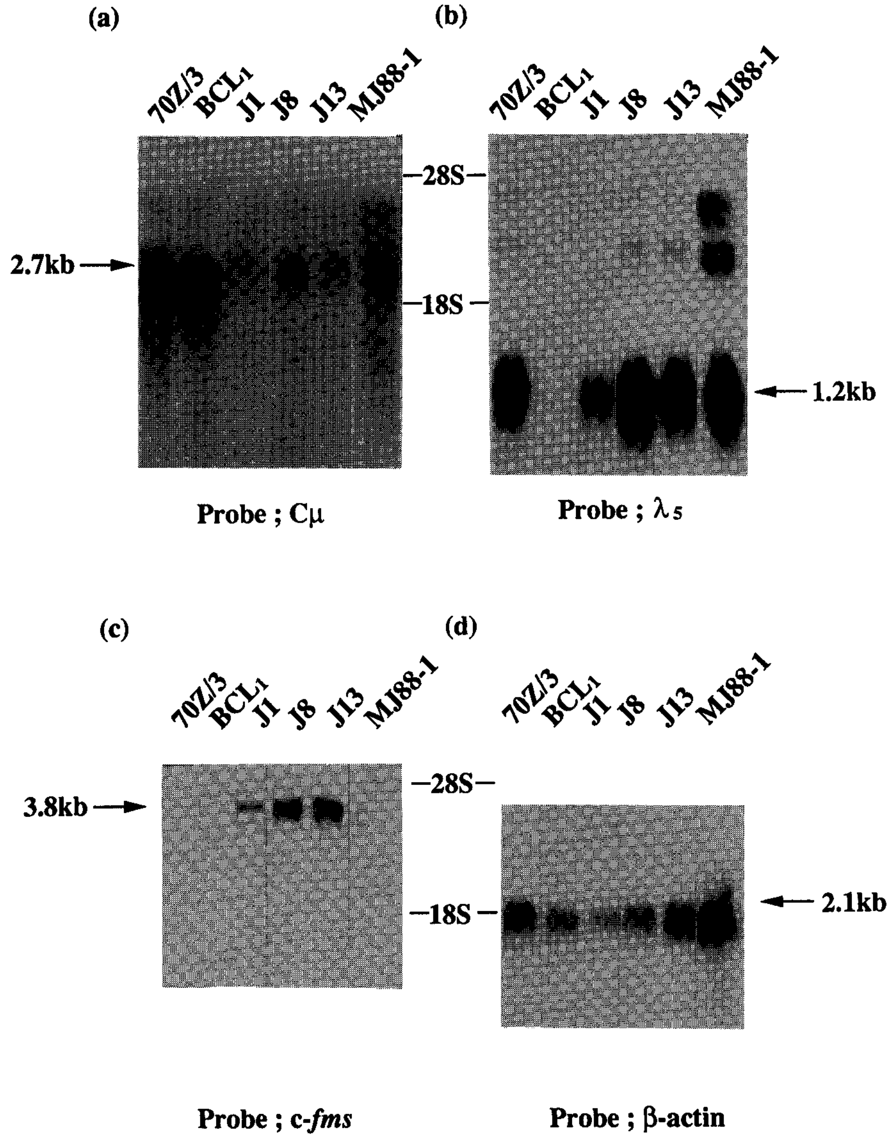

FIGURE 5. Expression of mRNA in pre-B cell lines. Total RNAs were prepared from various pre-B cell lines and a B-cell leukemia ($BCL_1$-B20), and were analyzed by Northern blot using $^{32}$P-labeled probes: (A) C*μ*, (B) *λ*5, (C) c-*fms*, or (D) *β*-actin as probes.

vation compatible with the slower proliferation of normal, or cultured, macrophages than early B-lineage cells. They had nearly 2–3 times the mean cell volume of their lymphoid counterparts, and adhered to culture flasks to varying degrees.

To examine the effect of IL-5 on the conversion of pre-B cells into macrophages by GM-CSF, we cultured J13 cells on ST2 layers with GM-CSF in combination with various amounts of IL-5 (0.5 to 50 U/ml). IL-5 inhibited conversion of pre-B cells by GM-CSF into macrophages in a dose-dependent manner. Limiting dilution analysis revealed that the frequency of conversion of the pre-B cell line J13 by GM-CSF into macrophages (J13GM) was approximately 1/500 and was less than 1/20,000 when IL-5 (50 U/ml) was added with GM-CSF. Attempts to regenerate B-lineage cells from macrophages by IL-5 were unsuccessful.

### DISCUSSION

We have shown here that nontransformed Ly-1$^+$ early B-lineage cell lines of the J series can be converted to Ly-1$^+$ macrophages under the influence of 5-azacytidine or GM-CSF. The four cloned cell lines that underwent this dramatic conversion included two at the early B precursor stage (J10 and J2) and two at the pre-B stage (J13 and J1).

IL-5 can support the growth of early B cells on

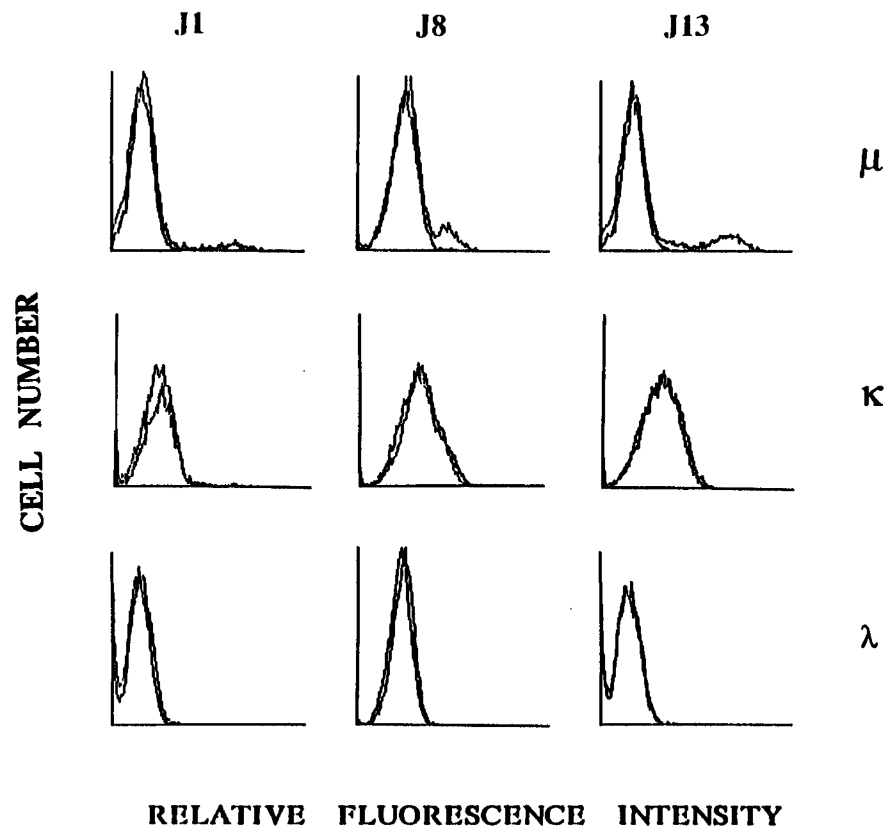

FIGURE 6. Expression of $\mu$-chain, but not $\kappa$- or $\lambda$-chain on the surface of pre-B cell lines. Pre-B cell lines were stained with FITC-coupled anti-$\mu$, FITC-coupled anti-$\lambda$ antibody, or phycoerythrin (PE)-coupled anti-$\kappa$ antibody. Cells stained were analyzed by a flow cytometer.

ST2 in LTBC-W after depleting mature B cells from bone marrow cells by Dexter-type LTBC (Tominaga et al., 1989). However, myeloid cells in addition to lymphoid cells also grow on ST2 in the system described before. As we reported (Tominaga et al., 1989) and describe in this study, Ly-1$^+$ B-lineage cells consist of a distinct population in the non-adherent fraction of 5-week LTBC-W of bone marrow cells from 3-week-old mice in the absence of IL-5. Most of early B-cell lines grown in LTBC-W, however, died when they reached certain cell numbers, usually from hundreds to several thousand in the absence of IL-5. As assessed by limiting dilution analysis for the cell growth, only about 0.1% of those cells continued to grow on the ST2 layer in the presence of IL-5. We thus estab-

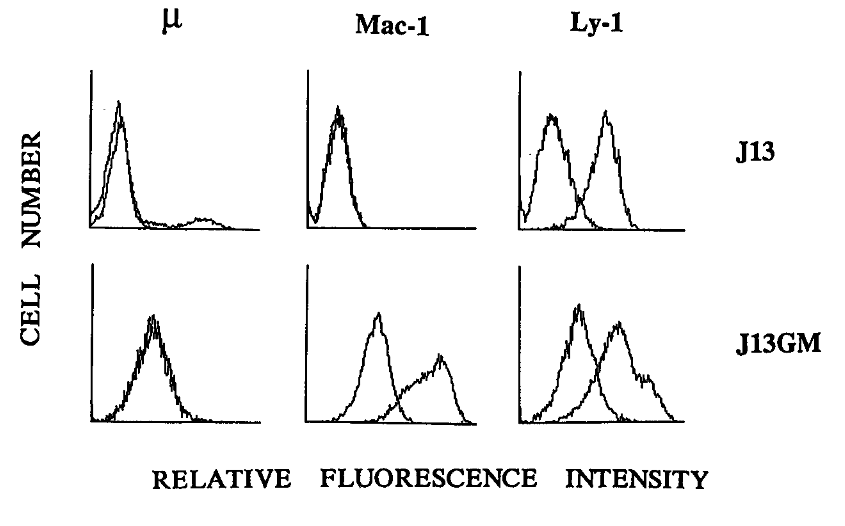

FIGURE 7. Phenotype characteristics of the macrophage-converted clone. Pre-B cell line (J13) or its macrophage-converted cell line (J13GM) by the treatment with GM-CSF for 5 weeks was stained by FITC-coupled anti-$\mu$, FITC-coupled anti-Ly-1, or anti-Mac-1 plus anti-rat $\kappa$. Cells stained were analyzed by a flow cytometry.

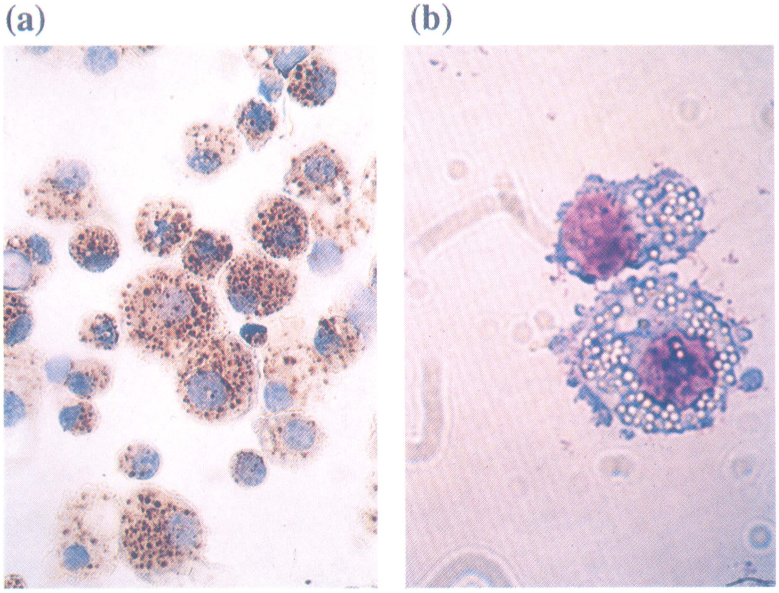

FIGURE 8. Morphological and cytochemical analysis of the macrophage-converted clone. Part (A) shows the converted J13 cells stained with nonspecific esterase, and (B) demonstrates their relative ability to phagocytose latex beads. The magnifications in (A) and (B) were 400× and 1000×, respectively. (See Colour Plate V at the back of this publication.)

lished IL-5-dependent Ly-1$^+$ B-lineage cells in the presence of IL-5 and ST2 stromal cells. Early B-lineage cell lines established in this study responded to IL-3 as well and IL-7 to a lesser extent. However, it did not respond to IL-1, IL-2, or IL-4. The supernatant from ST2 cells did not replace ST2 cells, strongly suggesting that cell-to-cell contact between early B-lineage or pre-B cells and ST2 was necessary to maintain growth.

It has been reported that B-cell precursors that develop in LTBC-W without exogenous IL-3 did not seem to give rise to Ly-1$^+$ early B cells (Dasch and Jones, 1986; Palacios and Leu, 1986; Palacios et al., 1987). There are at least two possibilities to account for the amazingly high incidence of Ly-1$^+$ B-lineage cells in our culture system. First, it may be related to the age of bone marrow donors used on starting materials for LTBC-W, because precursors of Ly-1$^+$ B cells exist in fetal liver, but are rare in adult bone marrow (Hayakawa et al., 1985). Second, it may have been caused by IL-5 itself. As shown in Figure 1A, Ly-1$^+$ cells were preferentially detectable when we cultured bone marrow cells from 3-week-old, but not from 8-week-old DBA/2J mouse. Ly-1$^+$ B lineage cells were also enriched in the period of culture only in the presence of IL-5 (Fig. 1B). We postulate that there may be precursor cells in bone marrow of Ly-1$^+$ B-lineage cells. When IL-5-independent and ST2-dependent pre-B-lineage cells (MJ88-1: sIgM$^-$, cIgM$^+$, Mac-1$^-$, Ia$^-$, Thy-1$^-$, IL-2R(Tac)$^-$, CD45R$^+$, and IL-5R$^-$) were cultured with IL-5 on the ST2 layer, expression of Ly-1 antigens remained negative (unpublished observations), suggesting that IL-5 does not induce expression of Ly-1 antigen on Ly-1$^-$ early B-lineage cells.

Ly-1$^+$ pre-B cell lines analyzed in this study expressed mRNA for membrane-type $\mu$-chain and $\lambda5$ (Fig. 5). MJ88-1 also expressed $\lambda5$ mRNA. In contrast, the T-88 cell line that grows in an IL-5-dependent and ST2-independent manner did not express $\lambda5$ mRNA (unpublished observation), suggesting that $\lambda5$ mRNA expression might correlate to stromal-cell dependency rather than IL-5 dependency. A striking feature of cell lines of the J series is that they express higher levels of c-*fms* mRNA compared with the MJ88-1 line (Fig. 5). The c-*fms* expression was also observed at the pro-B stage in the J series lines (data not shown), suggesting an earlier commitment of these Ly-1$^+$-lineage cells to be able to differentiate into macrophages.

Our data demonstrate unequivocally that normal Ly-1$^+$ B-lineage cell lines can be converted into macrophages by the treatment with 5-azacytidine or GM-CSF (Figs. 2, 3, 5, 7, and 8). The myeloid derivatives were adherent and phagocytic and had the morphology and size of macrophages (Figs. 3 and 8). They were independent of 2-ME, and displayed myeloid surface markers Mac-1 (Figs. 2 and 7), Mac-2, and F4/80 (Austyn and Gordon, 1981). Indeed, in every respect tested, they were indistinguishable from mature macrophages. Ly-1$^-$ pre-B cell lines did not convert to macrophages in some circumstances, including the addition of GM-CSF or 5-azacytidine (data not shown), suggesting that B-lineage cells maintained on ST2 in the presence of IL-5 may have distinct potential from those maintained in the absence of IL-5. It was reported that exposure of the pre-B lymphoma ABLS 8.1 to 5-azacytidine induced derivation of macrophagelike lines (Boyd and Schrader, 1982), and 5-azacytidine induced differentiation of IL-3-dependent cells into mature B cells (Palacios et al., 1987; Kinashi et al., 1988). Taken all together, DNA demethylation and gene activation of early B-lineage cells by 5-azacytidine may be one mechanism to control conversion to macrophage differentiation (Jones, 1985).

Thus, cells well along the Ly-1$^+$ B-lineage differentiation pathway acquired many features associated with the last stages of myeloid differentiation. It might be argued that GM-CSF did not induce these dramatic changes, but instead rescued rare spontaneous transformants. This seems to be unlikely in the case of GM-CSF, because GM-CSF induced the differentiation of Ly-1$^+$ B-lineage cells to macrophages only with the reduced rate of growth. Another possibility is the involvement of autocrine factor production, which may occur normally at some stage of myeloid differentiation. We cannot rule out this possibility at this time. The fact that IL-5 shows no significant effect on 5-azacytidine-induced conversion of Ly-1$^+$ early B-cell precursors to macrophages appears to be contradictory to the observation that IL-5 inhibits the conversion of Ly-1$^+$ pre-B cells to macrophages in response to GM-CSF. IL-5 might inhibit the signal transduction of GM-CSF. The effect of 5-azacytidine on B-lineage cells may be irrelevant with that of IL-5, because this drug affects the DNA directly.

Although we provide here the first evidence that treatment of nontransformed early B precursor cells with 5-azacytidine or GM-CSF, and nontransformed pre-B cells with GM-CSF provoke this switch, other lymphoid cell lines that could produce macrophages have been described previously (Boyd and Schrader, 1982; Bauer et al., 1986; Holmes et al., 1986; Seremetis et al., 1987; Davidson et al., 1988; Klinken et al., 1988). HAFTL-3, v-*ras* transformed pre-B lines, spontaneously generated subpopulations of Mac-1 expressing cells (Davidson et al., 1988), and the macrophage line P388D1 shares immunoglobulin rearrangements with the lymphoblastic line P388 derived from the same tumor (Bauer et al., 1986). Furthermore, switching to the myeloid lineage occurred frequently when B-lineage cells, from either lymphomas or preleukemia bone marrow cells of E$\mu$-*myc* transgenic mice, were infected with a retrovirus bearing v-*raf* (Klinken et al., 1988). The B-cell/macrophage switch might occur either by regression to a putative common lymphoid/myeloid precursor or by direct adoption of the macrophage differentiation program. Although most of our results are compatible with either explanation, it is noteworthy that cells of other hemopoietic lineages have not been observed in these experiments. Since granulocytes and macrophage diverge in myeloid differentiation (Kincade, 1987), the absence of cells of granulocyte morphology tends to favour direct switching to the mature of immature macrophage phenotype.

In summary, cell lines characteristic of nontransformed B-lineage cells are obtained from cells cocultivated with a stromal-cell layer and IL-5. In addition, four cell lines were found to co-express antigens usually restricted to the B-cell or myeloid pathways of differentiation. Detailed analysis suggest that the initial cells giving rise to these lines derived from a precursor common to the B-cell and myeloid lineages. IL-5 may only be required for supporting survival of the Ly-1$^+$ B-cell/macrophage progenitors, and M-CSF and/or GM-CSF appears to be required for conversion rather than growth support of spontaneously generated macrophages.

### MATERIALS AND METHODS

### Mice

DBA/2J mice were purchased from CLEA Japan Inc. (Tokyo). C3H/HeN IL-5 transgenic mice were produced (Tominaga et al., 1990) and were maintained in the animal facility of our university.

### Cytokines

Recombinant mouse IL-5 [rIL-5, $2.2 \times 10^{10}$ units (U)/g protein] was purified from cultured supernatant of IL-5 cDNA-transfected Chinese hamster ovary (CHO) cells according to the procedures previously described (Harada et al., 1987). Human rIL-1 ($2.0 \times 10^{10}$ U/g) and human rIL-2 ($4.3 \times 10^{10}$ U/g) were kind gifts by Dr. Y. Hirai (Tokushima Research Institute, Ohtsuka Pharmaceutical Co., Japan) and Dr. A. Kakinuma (Central Research Division, Takeda Chemical Industries Ltd., Osaka, Japan), respectively). Murine rIL-3 ($1.0 \times 10^{11}$ U/g) and murine IL-4 ($1.0 \times 10^{11}$ U/g) were kindly provided by Dr. K. Arai (DNAX Research Institute, Palo Alto, CA) and Dr. K. Hama (Ono Central Research Laboratory, Osaka, Japan), respectively. Murine rIL-7 ($4.0 \times 10^{12}$ U/g) (Sudo et al., 1989), murine rGM-CSF, and murine rIL-6 were generous gifts from Dr. T. Sudo (Biomaterial Research Center, Kamakura). We used ten-times concentrated supernatants as a M-CSF source of L cells (Kincade et al., 1979).

### Reagent and Antibodies

The following monoclonal antibodies were used for the surface staining: fluorescein isothiocyanate (FITC)-conjugated anti-Ly-1 (clone 53-7.3) and anti-Thy-1, and phycoerythrin (PE)-conjugated anti-mouse $\kappa$ antibodies (Becton-Dickinson, Mountain View, CA); FITC-conjugated anti-I-$A^d$ (Meiji Institute of Health Science, Tokyo); FITC-conjugated anti-IgM antibody (LO-MM-9) (Serotec Ltd., Blackthorn, Bicester, England); FITC-labeled anti-IgL (lambda, 1+2) (PharMigen, San Diego, CA); anti-Mac-2 (Boehringer Mannhein, West Germany); and FITC-labeled anti-rat $\kappa$-chain (MAR18.5) mAb, anti-IL-2R (7D4) mAb, and goat anti-mouse $\mu$-chain antibodies (prepared according to the procedures previously described (Riggs et al., 1958); and supernatants containing anti-Mac-1 M1/70 (Springer et al., 1979), anti-CD45R (RA3-6B2) (Coffman and Weissman, 1981), and anti-Lyb-2.1 (clone 9-6.1) (Southern, 1975) (kindly provided by Dr. H. Yakura, Tokyo Metropolitan Institute for Neuroscience, Tokyo, Yakura et al., 1981). 5-azacytidine was purchased from Sigma Fine Chemicals (St. Louis, MO).

### Culture and Cell Lines

RPMI 1640 medium and FCS were from Sigma and Microbiological Associates (Walkersville, MD), respectively. The complete medium described by Whitlock and Witte was used throughout this study that is, RPMI 1640 supplemented with 5% FCS, 50 $\mu$M 2-ME, 50 $\mu$g/ml streptomycin, and 50 U/ml penicillin. ST2 stromal-cell line (Ogawa et al., 1988) was kindly provided by Dr. S.-I. Nishikawa (Kumamoto University Medical School). IL-5- and ST2-dependent cell lines have been established according to procedures as previously described (Tominaga et al., 1989). In brief, we started from the original LTBC-W of 3-week-old mice. After 5 weeks, $2 \times 10^5$ nonadherent cells were transferred onto the ST2 layer and cultured in the presence of rIL-5 (50 U/ml). Two weeks later, 30 colonies were found in a well, and cells from each colony were transferred into a 25-$cm^2$ tissue-culture flask (SH-FLO25SW; TERUMO, Tokyo) with fresh ST2 layer and maintained for 3 months in the presence of IL-5. Eventually, we established 18 clones that were further analyzed in these studies. Cells growing on ST2 were fed with the medium for LTBC-W containing IL-5 (50 U/ml) twice a week. Cloning of cell lines was carried out by a limiting dilution technique using a flat-bottomed 96-well plate (Corning) with a ST2 cell layer in the presence of IL-5. A MJ88-1 cell line was established at the same time by the same procedures except that cells were cultured in the absence of IL-5. Growth of MJ88-1 is ST2-dependent and IL-5-independent.

### Induction of Differentiation

Induction of differentiation of B cells by using 5-azacytidine was carried out according to methods originally described by Boyd and Schrader (1982) with slight modifications. In brief, early B-cell clones [$5 \times 10^6$/ml in 4 ml of IL-5 (50 U/ml)-containing medium] were exposed in a 25-$cm^2$ flask (TERUMO, Tokyo) to several concentrations of freshly prepared 5-azacytidine (from 2 to 20 $\mu$g/ml) at 37°C for 24 h. After this step, 4 ml of IL-5-containing medium without 5-azacytidine was added to the culture and incubated at 37°C. After 3 days, the cells were harvested, washed twice in the medium, and the viable cells (approximately 10% of the starting population) were co-cultured on the ST2 layer at $2\text{–}3 \times 10^5$ cells/ml with medium containing IL-5. The culture medium was changed into fresh IL-5 containing medium twice a week. Nonadherent cells were harvested and monitored for the presence of $\mu^+$, Ly-$1^+$, CD45$R^+$, and Mac-$1^+$ cells for 5 weeks after exposures to the drug.

Pre-B cells ($2 \times 10^6$) were co-cultured with ST2 in 25-$cm^2$ flasks in a final volume of 7-ml culture medium in the presence of various cytokines: GM-CSF (100 U/ml), M-CSF (10% of LCCM), IL-4 (100 U/ml), IL-5 (50 U/ml), or IL-7 (100 U/ml). The cultures were monitored for the presence of Mac-$1^+$ or sIg$M^+$ cells 14, 21, and 40 days after exposure to cytokines.

### Limiting Dilution Analysis

Limiting dilution analysis was carried out as described in Methods (Ogawa et al., 1988). Cells (10 to 10,000 per well with a threefold dilution) were co-cultured with IL-5 (50 U/ml) and GM-CSF (100 U/ml), or in the presence of GM-CSF alone on the ST2 layer for 4 weeks.

### Immunofluorescence Staining and Flow Cytometry Analysis

Expression of cell-surface antigen was analyzed by flow cytometry as previously described (Tominaga et al., 1989). Cells ($1 \times 10^6$) were stained directly with 50 μl of FITC- or PE-conjugated antibody (20 μg/ml) for 20 min on ice and washed three times with 5% FCS-PBS. In some cases, $1 \times 10^6$ cells were incubated with 50 μl of antibodies (20 μg/ml) for 20 min on ice. After washing once, the cells were then stained with 50 μl of FITC-conjugated anti-rat κ-light chain antibody (MAR18.5) (20 μg/ml). For analysis, EPICS V (Coulter Co., Hialeah, FL) was used and only the cells within the lymphocyte gate were counted as described (Tominaga et al., 1989).

### Morphological Determination of Cultured Cells

Cytocentrifuge preparations of cells were stained with May–Grunwald and Giemsa solution (Merck, Darmstadt, FRG). All the cells that had polymorphic nucleus were counted as polymorphonuclear (PMN) cells. The rest of the cells were further classified into mononuclear cells of small, medium, and large size. In this classification, particularly for the cells in LTBC-W, all small mononuclear cells are actually small lymphocytes, but large- and medium-size mononuclear cells may include immature cells of other hemopoietic cell lineages as well as lymphoid cell lineage.

### Assays for Macrophage Characteristics

Nonspecific esterase granules were detected by the α-naphtylbutyrate (Sigma) method (Li et al., 1973). Phagocytic activity was determined at 37°C by adding 1 μm latex beads (Sigma) to the cultures and by examining the cultures 3 h later; cells were collected, cytocentrifuged, and stained with May–Grunwald–Giemsa, and the percentage of nonspecific esterase-positive cells that had taken up beads was scored at 400 × magnification.

### Southern Blot Analysis

Preparation of high molecular weight DNA, Southern blot analysis, and hybridization with the $J_H$ probe were carried out according to the methods previously described (Southern, 1975; Tominaga et al., 1989). In brief, 5 μg of DNA from cloned cell lines and DBA/2J liver cells were digested to completion with *Eco*RI or *Bam*HI (Toyobo Co. Ltd., Osaka, Japan), then separated on 0.8% agarose gels and transferred to nylon membrane filters (Gene Screen Plus, NEN-Du Pont, NJ) for hybridization. Southern blots were hybridized with $^{32}P$-labeled DNA probes at 42°C in a buffer containing 50% formamide, 1% SDS, 1 M NaCl, 10% dextran sulfate, and 250 μg/ml salmon sperm DNA. The $^{32}P$-labeled probes were labeled using a Oligolabeling Kit (Pharmacia Fine Chemicals, Uppsala, Sweden). Bands that hybridized with labeled probes were detected by autoradiography with RXO-H film (Fuji Photo Film Co. Ltd., Tokyo, Japan) and Du Pont intensifying screen. The probes used were a $J_H4$ probe, 2.6 kb *Hind*III-*Bam*HI fragment (Sakano et al., 1981) containing $J_H4$ gene (a generous gift of Dr. T. Honjo, Kyoto University); $J_\kappa$, a 1.6 kb *Hind*III-*Xba*I fragment (Sakano et al., 1979) of Bluescript-KS containing $J_{\kappa1}$-$J_{\kappa5}$ gene (a kind gift from Dr. Y. Kurosawa, Fujita Medical College).

### RNA Analysis

RNA was prepared according to the described methods using the acid guanidinium thiocyanate-phenol-chloroform method (Chomczynski and Sacchi, 1987). The methods used for Northern blotting are essentially the same as those described by Lehrach et al. (1977). Twenty micrograms of RNA were electrophoresed through 1.5% agarose horizontal gels in 1 × MOPS buffer (0.2 M morpholinopropanesulfonic acid sodium salt, 0.05 M sodium acetate, 0.01 M $Na_2EDTA$ (pH is adjusted to 7.0 with acetic acid) (pH 7.0), and 6% formamide at 20 to 30 V for 16 h, and directly transferred to Gene Screen membrane (NEN-Du Pont, NJ). The blot was baked in a vacuum oven for 3 h at 80°C. The RNA blot was prehybridized for 8 to 20 h at 42°C in the prehybridization buffer, which contains 50% (vol/vol) formamide, 50 mM Tris-HCl (pH 7.5), 1 M NaCl, 0.1% sodium pyrophosphate, 1% SDS, denatured salmon sperm DNA at 250 μg/ml, 10% dextran sulfate, and 0.2% each of bovine serum albumin, Ficoll, and polyvinylpyrrolidone. After incubation of the RNA blots of 42°C with $5 \times 10^7$ CPM of $^{32}P$-labeled DNA fragments in the same buffer, the filters were rinsed with two changes of 0.5×SSC for 5 min each at room temperature and then washed with two changes of 0.5 ×SSC-1% SDS for 15 min each at 65°C followed by two changes of 0.1 ×SSC at room temperature. Kodak XAR-5 X-ray film was exposed to the filters for 1 to 3 days at −80°C, using intensifying screens to obtain autoradiographs. The $^{32}P$-labeled DNA probes used were the following: C$\mu$ probe, the 1.6-kb *Hae* II fragment from $\mu$-heavy-chain constant region-specific cDNA plasmid (pAB$\mu$-1) (Alt et al., 1980) that includes most $C_H3$ and entire $C_H4$ domains, kindly provided by Dr. Alfred Bothwell (Yale University, New Haven, CN); $\lambda$5 probe, a 0.26 kb *Eco*RI fragment of pZ183-1a (Sakaguchi and Melchers, 1986; Kudo et al., 1987); and v-*fms*, a 1.4 kb *Pst*I fragment of feline sarcoma virus (Donner et al., 1982) (from American Tissue Culture Collection, Rockville, MD); and $\beta$-actin, 0.33 kb *Eco*RI fragment of pMA$\beta$-3′ untranslated region (Tokunaga et al., 1986) (kindly provided by Dr. K. Tokunaga, Chiba Cancer Research Institute, Chiba, Japan).

### ACKNOWLEDGMENTS

We are grateful to Drs. Y. Hirai, A. Kakinuma, K. Arai, K. Hama, and T. Sudo for providing recombinant interleukins, to Dr. Honjo for $J_H$ probe, Dr. Y. Kurosawa for $J_\kappa$ probe, and to Drs. S.-I. Nishikawa and H. Yakura for mAbs. We are also indebted to Dr. P. Kincade for his critical reading of this manuscript. This work was supported in part by Grant-in-Aid for Cancer Research and for Scientific Research from the Japanese Ministry of Education, Science, and Culture; by the research grant of the Ichiro Kanehara Memorial Foundation, and by a grant from the Pharmaceutical Foundation.

*(Received March 5, 1990)*

*(Accepted June 30, 1990)*

### REFERENCES

Alt F.W., Bothwell A.L.M., Knapp M., Siden E., Mather E., Koshland M., and Baltimore D. (1980). Synthesis of secreted and membrane-bound immunoglobulin Mu heavy chains is directed by mRNAs that differ at their 3′ ends. Cell **20:** 293–301.

Austyn J.M., and Gordon S. (1981). F4/80, a monoclonal antibody directed specifically against the mouse macrophages. Eur. J. Immunol. **11:** 805–815.

Bauer S.R., Holmes K.L., Morse H.C., III, and Potter M. (1986). Clonal relationship of the lymphoblastic cell line P388 to the macrophage cell line P388D1 as evidenced by immunoglobulin gene rearrangements and expression of cell surface antigens. J. Immunol. **136:** 4695–4699.

Boyd A.W., and Schrader J.W. (1982). Derivation of macrophage-like lines from the pre-B lymphoma ABLS8.1 using 5-azacytidine. Nature **297:** 691–693.

Chomczynski P., and Sacchi N. (1987). Single step method of RNA isolation by acid guanidinium thiocyanate-phenol-chloroform extraction. Ann. Biochem. **162:** 156–159.

Coffman R.L., and Weissman I.L. (1981). B220: A B cell-specific member of the T200 glycoprotein family. Nature **289:** 681–683.

Coffman R.L., Seymour W.P., Lebman D.A., Hiraki D.D., Christiansen J.A., Shrader B., Cherwinski H.M., Savelkoul H.F.J., Finkelman F.D., Bond M.W., and Mosmann T.R. (1988). The role of helper T cell products in mouse B cell differentiation and isotype regulation. Immunol. Rev. **102:** 5–28.

Coffman R.L., Seymour W.P., Hudak S., Jackson J., and Rennick D. (1989). Antibody to interleukin-5 inhibits helminth-induced eosinophilia in mice. Science **245:** 308–310.

Dasch J.R., and Jones P.P. (1986). Independent regulation of IgM, IgD, and Ia antigen expression in cultured immature B lymphocytes. J. Exp. Med. **163:** 938–951.

Davidson W., Pierce J.H., Rudikoff S. and Morse H.C., III. (1988). Relationships between B cell and myeloid differentiation. Studies with a B lymphocyte progenitor line, HAFTL-1. J. Exp. Med. **168:** 389–407.

Donner L., Fedele L.A., Garon C.F., Anderson S.J., and Sherr C.J. (1982). McDonough feline sarcoma virus: Characterization of the molecularly cloned provirus and its feline oncogene (v-*fms*). J. Virol. **41:** 489–500.

Harada N., Takahashi T., Matsumoto M., Kinashi T., Ohara J., Kikuchi Y., Koyama N., Severinson E., Yaoita Y., Honjo T., Yamaguchi N., Tominaga A., and Takatsu K. (1987). Production of a monoclonal antibody useful in the molecular characterization of murine T cell-replacing factor/B cell growth factor II. Proc. Natl. Acad. Sci. USA **84:** 4581–4585.

Hayakawa K., Hardy R.R., Herzenberg L.A., and Herzenberg L.A. (1985). Progenitors for Ly-1 B cells are distinct from progenitors for other B cells. J. Exp. Med. **161:** 1554–1568.

Holmes K.L., Pierce J.H., Davidson W.F., and Morse H.C., III. (1986). Murine hematopoietic cells with pre-B or pre-B/myeloid characteristics are generated by *in vitro* transformation with retroviruses containing *fes*, *ras*, *abl*, and *src* oncogenes. J. Exp. Med. **164:** 443–457.

Jones P.A. (1985). Effects of 5-azacytidine and its 2′-deoxyderivative on cell differentiation and DNA methylation. Pharmac. Ther. **28:** 17–27.

Kinashi T., Harada N., Severinson E., Tanabe T., Sideras P., Konishi M., Azuma C., Tominaga A., Bergstedt-Lindqvist S., Takahashi M., Matsuda F., Yaoita Y., Takatsu K., and Honjo T. (1986). Cloning of complementary DNA encoding T-cell replacing factor and identity with B-cell growth factor II. Nature **324:** 70–73.

Kinashi T., Inaba K., Tsubata T., Tashiro K., Palacios R., and Honjo T. (1988). Differentiation of an interleukin-3-dependent precursor B-cell clone into immunoglobulin-producing cells in vitro. Proc. Natl. Acad. Sci. USA **85:** 4473–4477.

Kincade P.W. (1987). Experimental models for understanding B lymphocytes formation. Adv. Immunol. **41:** 181–267.

Kincade P.W., Lee G., Fernandes G., Moore M.A.S., Williams N., and Good R.A. (1979). Abnormalities in clonable B lymphocytes and myeloid progenitors in autoimmune NZB mice. Proc. Natl. Acad. Sci. USA **76:** 3464–3468.

Klinken S.P., Alexander W.S., and Adams J.M. (1988). Hemopoietic lineage switch: v-*raf* oncogene converts E$\mu$-*myc* transgenic B cells into macrophages. Cell **53:** 857–867.

Kudo A., Sakaguchi N., and Melchers F. (1987). Organization of the murine Ig-related $\lambda$5 gene transcribed selectively in pre-B lymphocytes. EMBO J. **6:** 103–107.

Lehrach H., Diamond D., Wozney J.M., and Boedtker H. (1977). RNA molecular weight determinations by gel electrophoresis under denaturing conditions, a critical re-examination. Biochemistry **16:** 4743–4751.

Li C.Y., Lam K.W., and Yam L.T. (1973). Esterases in human leukocytes. J. Histochem. Cytochem. **21:** 1–12.

Migita M., Yamaguchi N., Katoh S., Mita S., Matsumoto R., Sonoda E., Tsuchiya H., Matsuda I., Tominaga A., and Takatsu K., (In press). Elevated expression of c-*myc* proto-oncogene during interleukin-5-induced growth and differentiation of murine B lineage cells.

Ogawa M., Nishikawa S., Ikuta I., Yamamura F., Naito M., Takahashi K., and Nishikawa S.-I. (1988). B cell ontogeny in murine embryo studied by a culture system with the monolayer of a stromal cell clone, ST2: B cell progenitor develops first in the embryonal body rather than in the yolk sac. EMBO J. **7:** 1337–1343.

Palacios R., Karasuyama H., and Rolink A. (1987). $Lyl^+$ pro-B lymphocyte clones. Phenotype, growth requirements and differentiation *in vitro* and *in vivo.* EMBO J. **6:** 3687–3693.

Palacios R., and Leu T. (1986). CC11: A monoclonal antibody specific for interleukin-3-sensitive mouse cells defines two major populations of B cell precursors in the bone marrow. Immunol. Rev. **93:** 125–146.

Riggs J.L., Seiwald R.J., Burckhalter J.N., Downs C.W. and Metcalf T.G. (1958). Isothiocyanate compounds as fluorescent labeling agents for immune serum. Amer. J. Pathol. **34:** 1081–1097.

Sakaguchi N., and Melchers F. (1986). λ5, a new light-chain-related locus selectively expressed in pre-B lymphocytes. Nature **324:** 579–582.

Sakano H., Huppi K., Heinrich G., and Tonegawa S. (1979). Sequences at the somatic recombination sites of immunoglobulin light-chain genes. Nature **280:** 288–294.

Sakano H., Kurosawa Y., Weigert M., and Tonegawa S. (1981). Identification and nucleotide sequence of a diversity DNA segment (D) of immunoglobulin heavy chain genes. Nature **290:** 562–565.

Sanderson C.J., Campbell H.D., and Young I.G. (1988). Molecular and cellular biology of eosinophil differentiation factor (IL-5) and its effects on human and mouse B cells. Immunol. Rev. **102:** 29–50.

Sariban E., Mitchell T., and Kufe D. (1985). Expression of the c-*fms* proto-oncogene during human monocytic differentiation. Nature **316:** 64–66.

Seremetis S. V., Pelicci P.-G., Tabilio A., Ubriaco A., Grignani F., Cuttner J., Winchester R.J., Knowles II D.M., and Dalla-Favera R. (1987). High frequency of clonal immunoglobulin or T cell receptor gene rearrangements in acute myelogenous leukemia expressing terminal deoxyribonucleotidyl transferase. J. Exp. Med. **165:** 1703–1712.

Sherr C.J., Rettenmier C.W., Sacca R., Roussel M.F., Look A.T., and Stanley E.R. (1985). The c-*fms* proto-oncogene product is related to the receptor for the mononuclear phagocyte growth factor, CSF-1. Cell **41:** 665–676.

Southern E. (1975). Detection of specific sequences among DNA fragments separated by gel electrophoresis. J. Mol. Biol. **98:** 503–512.

Springer T., Galfre G., Secher D.S., and Milstein C. (1979). Mac-1: A macrophage differentiation antigen identified by monoclonal antibody. Eur. J. Immunol. **9:** 301–306.

Sudo T., Ito M., Ogawa Y., Iizuka M., Kodama H., Kunisada T., Hayashi S.-I., Ogawa M., Sakai K., Nishikawa S., and Nishikawa S.-I. (1989). Interleukin-7 production and function in stromal cell-dependent B cell development. J. Exp. Med. **170:** 333–338.

Takatsu K., Kikuchi Y., Takahashi T., Honjo T., Matsumoto M., Harada N., Yamaguchi N., and Tominaga A. (1987). Interleukin-5, a T-cell-derived B-cell differentiation factor also induces cytotoxic T lymphocytes. Proc. Natl. Acad. Sci. USA **84:** 4234–4238.

Takatsu K., Tominaga A., Harada N., Mita S., Matsumoto M., Takahashi T., Kikuchi Y., and Yamaguchi N. (1988). T cell-replacing factor (TRF)/interleukin 5 (IL-5): Molecular and functional properties. Immunol. Rev. **102:** 107–135.

Tokunaga K., Taniguchi H., Yoda K., Shimizu M., and Sakiyama S. (1986). Nucleotide sequence of a full-length cDNA for mouse cytoskeletal β-actin mRNA. Nucleic Acids Res. **14:** 2829.

Tominaga A., Takaki S., Koyama N., Matsumoto R., Kanai Y., Yamauchi S., Miyazaki J.-I., Yamamura K.-I., and Takatsu K. (1990). Physiological consequences of IL-5 transgene expression. *In:* The molecular biology of immune diseases and the immune response: Advances in gene technology (Proceedings of the Miami Winter Symposium on Biotechnology, Oxford, England, Oxford University Press.), p. 245.

Tominaga A., Mita S., Kikuchi Y., Hitoshi Y., Takatsu K., Nishikawa S.-I., and Ogawa M. (1989). Establishment of IL-5 dependent early B cell lines by long-term bone marrow cultures. Growth Factors **1:** 135–146.

Whitlock C.A., and Witte O.N. (1982). Long-term culture of B lymphocytes and their precursors from murine bone marrow. Proc. Natl. Acad. Sci. USA **79:** 3608–3612.

Yakura H., Shen F.-W., Kaemmer M., and Boyse E.A. (1981). Lyb-2 system of mouse B cells. Evidence for a role in the generation of antibody-forming cells. J. Exp. Med. **153:** 129–135.

Yokota T., Coffman R.L., Hagiwara H., Rennick D.M., Takebe Y., Yokota K., Gemmell L., Shrader B., Yang G., Meyerson P., Luh J., Hoy P., Pene J., Briere F., Spits H., Banchereau J., Vries J.D., Lee F.D., Arai N., and Arai K. (1987). Isolation and characterization of lymphokine cDNA clones encoding mouse and human IgA-enhancing factor and eosinophil colony-stimulating factor activities: Relationship to interleukin 5. Proc. Natl. Acad. Sci. USA **84:** 7388–7392.

